# Otoendoscopy in the era of narrow-band imaging: a pictorial review

**DOI:** 10.1007/s00405-022-07656-5

**Published:** 2022-09-21

**Authors:** Federica Pollastri, Luca Giovanni Locatello, Chiara Bruno, Giandomenico Maggiore, Oreste Gallo, Rudi Pecci, Beatrice Giannoni

**Affiliations:** 1 Unit of Audiology, Oncological and Robotic Head and Neck Surgery, Careggi University Hospital, Largo Brambilla 3, 50134 Florence, Italy; 2 Department of Neuroscience, Psychology, Drug’s Area and Child’s Health, University of Florence, Florence, Italy; 3 Department of Otorhinolaryngology, Careggi University Hospital, Florence, Italy

**Keywords:** Narrow-band imaging, Otoendoscopy, NBI, Otological diagnosis, External and middle ear pathologies

## Abstract

**Purpose:**

Otoendoscopy represents the initial non-invasive diagnostic cornerstone for external and middle ear disorders. Recently, new techniques of enhanced imaging such as narrow-band imaging (NBI) have been introduced but their role as a potential aid in otological practice remains unproven. In this pictorial review, we want to present the potential application of this endoscopic method, highlight its limitations, and give some hints regarding its future implementation.

**Methods:**

Representative cases of external and/or middle ear pathologies were selected to illustrate the role of NBI in this regard.

**Results:**

NBI may represent a useful aid in the otological work-up, in the differential diagnosis of ear tumor-like masses, and, possibly, in the prognosis of tympanic perforations. For other ear disorders, instead, this technique does not seem to add anything to the standard clinical practice.

**Conclusions:**

NBI might prove useful in the assessment of selected external and middle ear disorders but its role must be prospectively validated.

## Introduction

Otoendoscopy has gained much popularity in the last years both for diagnostic and surgical purposes [1, 2]. Compared to traditional microscopic techniques, otoendoscopy has several advantages including a wider and magnified field of view, and the availability of angled optics that allow to “look around the corner”; endoscopy also represents an extraordinary teaching tool [3, 4].

Recently several technical enhancements have been introduced in addition to standard white light (WL) endoscopy, mainly by the application of specific optical filters: for example, the Spectra A and Spectra B filters (the former reduces the red hues, while the latter enhances the green and blue spectral component; both from the IMAGE1 S, Karl Storz SE & Co,Tuttlingen, Germany) or the narrow band imaging technology (NBI, Olympus Medical, Tokyo, Japan), which highlights the microvascular texture using blue and green light wavelengths [5].

In the otological field, a recent publication has shown how the use of the former system revealed a sensitivity of 97% and a specificity of 95% in the identification of residual cholesteatoma that was confirmed histologically [6]. Instead, in a small series of sixteen cases of cholesteatoma surgery, NBI demonstrated no objective difference in the definition of epithelial borders compared to WL [7]. The NBI technique has been extensively used in the diagnosis and differentiation of premalignant and malignant lesions of the mucosa of the upper aerodigestive tract [8–11]; more recently, our group has shown how NBI might be useful also in differentiating vascularized middle ear masses, such as paragangliomas or aural polyps [12].

To further explore the role of this technique in the otoendoscopic diagnostic workup, we have carried out a critical and comprehensive evaluation of external and middle ear lesions by NBI.

## Methods

The present study was performed at the Department of Otorhinolaryngology and the Unit of Audiology of the Careggi University Hospital in Florence, Italy, during the period June 2021–March 2022. Otoendoscopy was performed using a flexible endoscope which was connected to a video processor (VISERA Elite OTV-S190) equipped with a xenon light source (VISERA Elite CLV-S190); the video monitor used was the OEV261H 26’’ LCD HD (all from Olympus Medical System Corporation, Tokyo, Japan). The Medicap system (Medicap USB300, MediCapture Inc., Plymouth Meeting, PA, USA) was used for recording and archiving high-definition and anonymized pictures on a hard-disk drive. White balance was always performed before the otoendoscopic registration.

Three authors with long-standing expertise in NBI endoscopy (FP, CB and LGL) performed the otoendoscopic examinations videos and collected the cases. NBI was used systematically on each patient requiring otoscopy during the study period and then cases were regrouped by each otological condition (cholesteatoma, osteoma, aural polyps, etc.). Cerumen removal was performed before the examination but pictures were excluded if the procedure had traumatized the external ear canal and/or had caused any bleeding. Cases were also discarded if image artifacts were present or if the pictures appeared blurry because of insufficient lighting or incorrect angulation. The remaining pictures were ultimately reorganized in folders and reviewed separately by each author. A description of the otoendoscopic appearance under WL and NBI was given for each condition by identifying the common features in terms of vascularization. For all the included cases, subsequent clinical and radiological work-up and the final diagnosis were retrieved from the digital records. Panels were created by the open source image editor GIMP (GNU Image Manipulation Program, The GIMP Development Team, Berkeley, California, USA, available at: https://www.gimp.org). Informed consent was obtained from all patients involved in this article and procedures were always conducted in accordance to the Helsinki declaration.

## Results

A total of 2160 patients were investigated by otoendoscopy, for a total of 4320 ears. After applying the selection criteria 180 ears have been analyzed, the most common conditions encountered being tympanic membrane perforation (16.7%), middle ear dysventilation (34.4%) and different forms of otitis (22.8%).

### Tympanic membrane perforation

Tympanic membrane perforations are commonly associated with infections (acute otitis media—AOM, chronic otitis media—COM), external trauma, or barotrauma; often, they heal spontaneously but sometimes tympanoplasty type I (myringoplasty) is required to restore the correct anatomical continuity [13]. We registered the pictures of 30 cases of unilateral tympanic perforation, 12 having an acute onset and 18 of long-standing origin. In the former group, 9 spontaneously healed within 6 months, while the remaining 3 cases, together with the 18 long-standing perforations, were all treated with surgery.

**Fig. 1:**
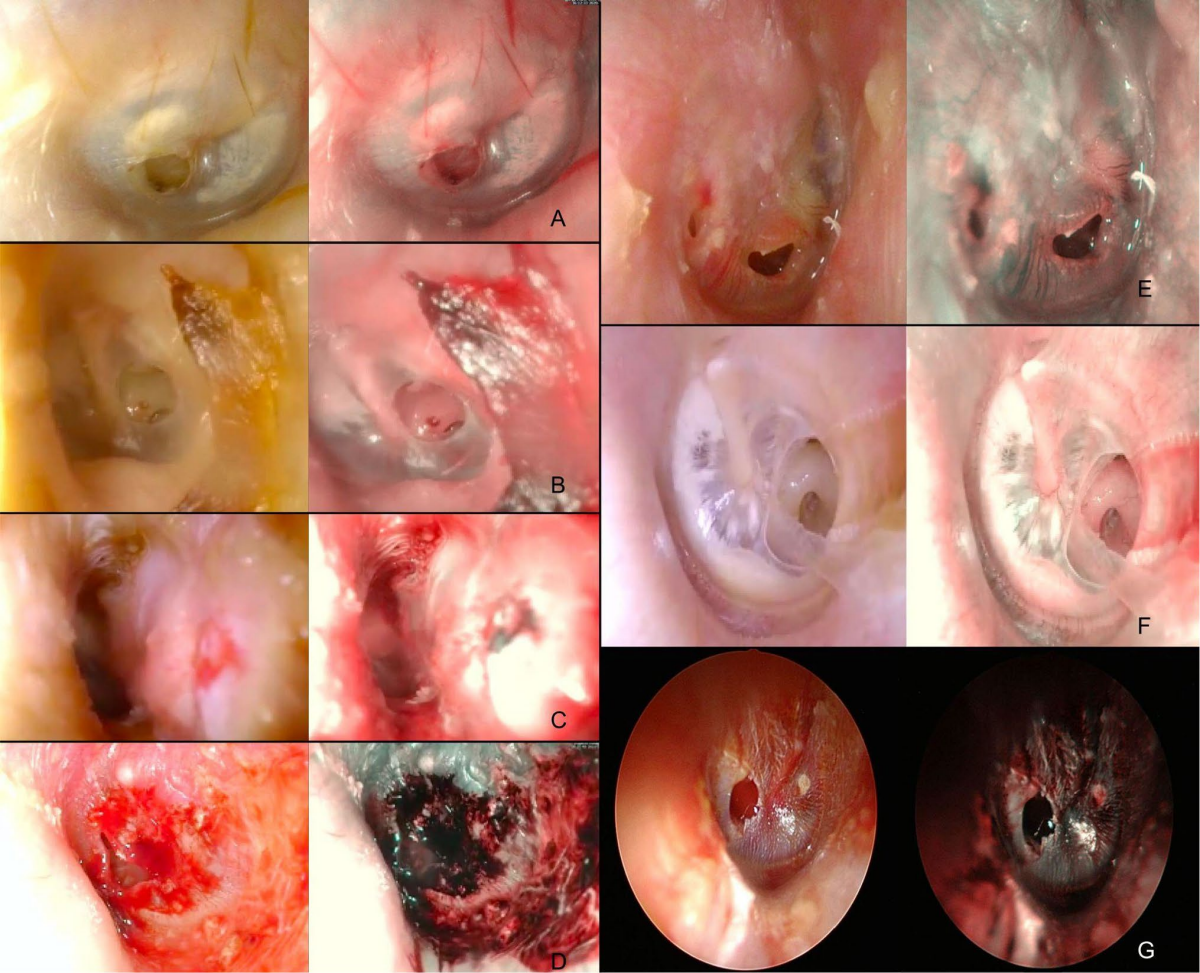
**A**, **B** Small tympanic perforations; **C** myringoplasty; **D**, **G** traumatic perforations; **E** post-AOM perforations; **F** perforation in the context of COM

Several vascular patterns could be identified according to the cause of perforation, the timing, and the healing process. When seen acutely, traumatic perforations (11/12 cases, 91,7%) showed a diffuse hyperemic vascularization around the eardrum defect, as well as involving the skin of the ear canal (Fig. 1D, G). Conversely, in the case of post-AOM perforations, the vasculature did not involve the borders (Fig. 1e). After some months, the now smaller perforations retained only a modest vascular texture around the edges (Fig. 1A, B).

Perforations in the context of COM typically showed a poorly represented vascularization (Fig. 1F) that instead was seen when a temporal fascia graft had been placed by myringoplasty (Fig. 1C).

### Atrophic areas, pars tensa retractions and myringosclerosis

We studied 25 patients with atrophic areas of the eardrum (whose origin was a previous perforation of various etiology in 14 and placement of a ventilation tube in 11), 23 cases of pars tensa retraction pocket (10 grade II according to Sadé-Berco classification, 7 grade III and 6 grade IV) [14] and 14 patients with myringosclerosis.

**Fig. 2:**
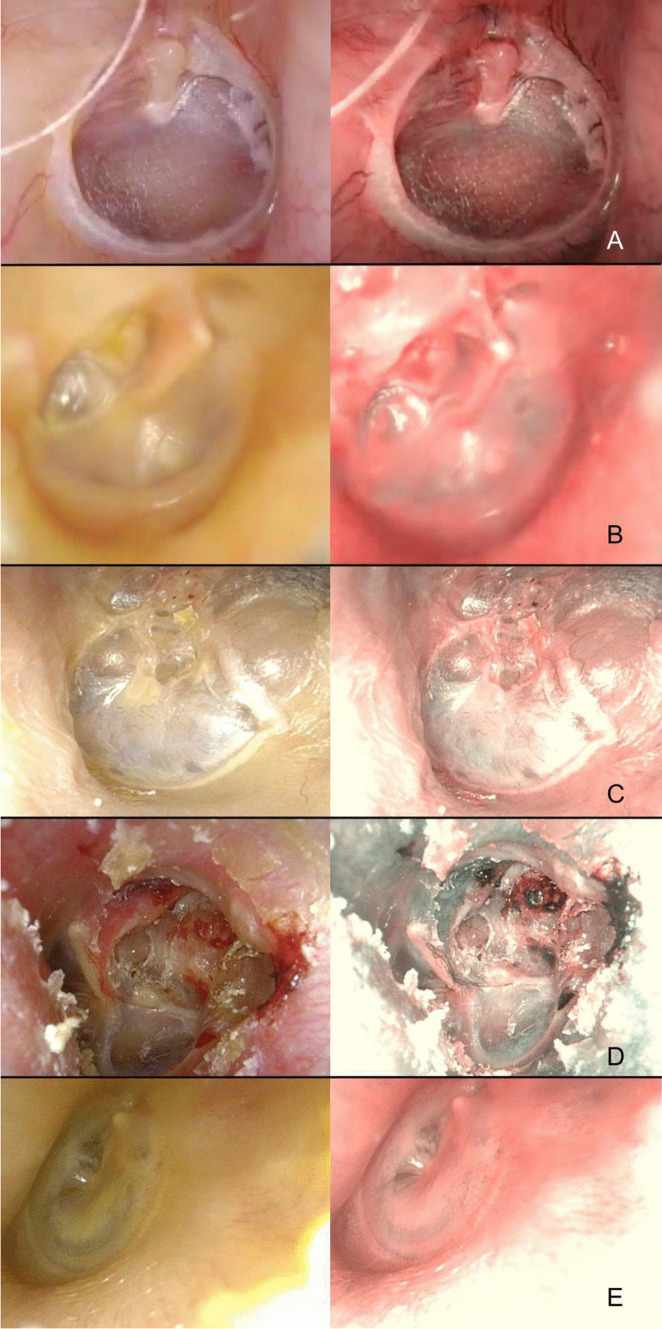
**A** Large atrophic area; **B** retraction pocket of the pars tensa at the incudo-stapedial joint region; **C**, **D** adhesive otitis media; **E** myringosclerosis

Tympanic membrane atrophy is a possible consequence of any middle ear disease (AOM with perforation, recurrent otitis media with effusion, ventilation tube placement) where there is a loss of the middle layer of the eardrum [15]. In 100% of the analyzed cases, NBI was completely negative in these atrophic areas (Fig. 2A).

Even for retraction pockets of the pars tensa and independently from the grade, no vascularization was ever apparent in the retracted areas (Fig. 2b) [16]. When the tympanic membrane is completely bound to the middle ear structures, such as the promontory or ossicular chain by fibrous adhesions, it is called adhesive otitis media (AdOM) [17]. Again, the use of the NBI method did not add information, because the fibrous tissue is NBI-negative (Fig. 2C, D).

Tympanosclerosis can be a consequence of AOM or a complication of myringotomy that leads to collagen and hyaline material deposition into the middle ear mucosa. Myringosclerosis, instead, is a type of tympanosclerosis characterized by calcification and sclerosis formation on the tympanic membrane [18]. Again, in all analyzed cases no vascularization was ever apparent under NBI in these calcified areas of the eardrum (Fig. 2E).

### Infectious external/middle ear disorders: bullous hemorrhagic myringitis, bacterial otitis externa, otomycosis, acute otitis media, and granular myringitis

With NBI methodology we evaluated several forms of external and/or otitis media, in particular 3 cases of bullous hemorrhagic myringitis, 15 bacterial otitis externa, 13 cases of otomycosis (3 of them with an associated tympanic perforation), 6 AOM and 2 granular myringitis.

**Fig. 3:**
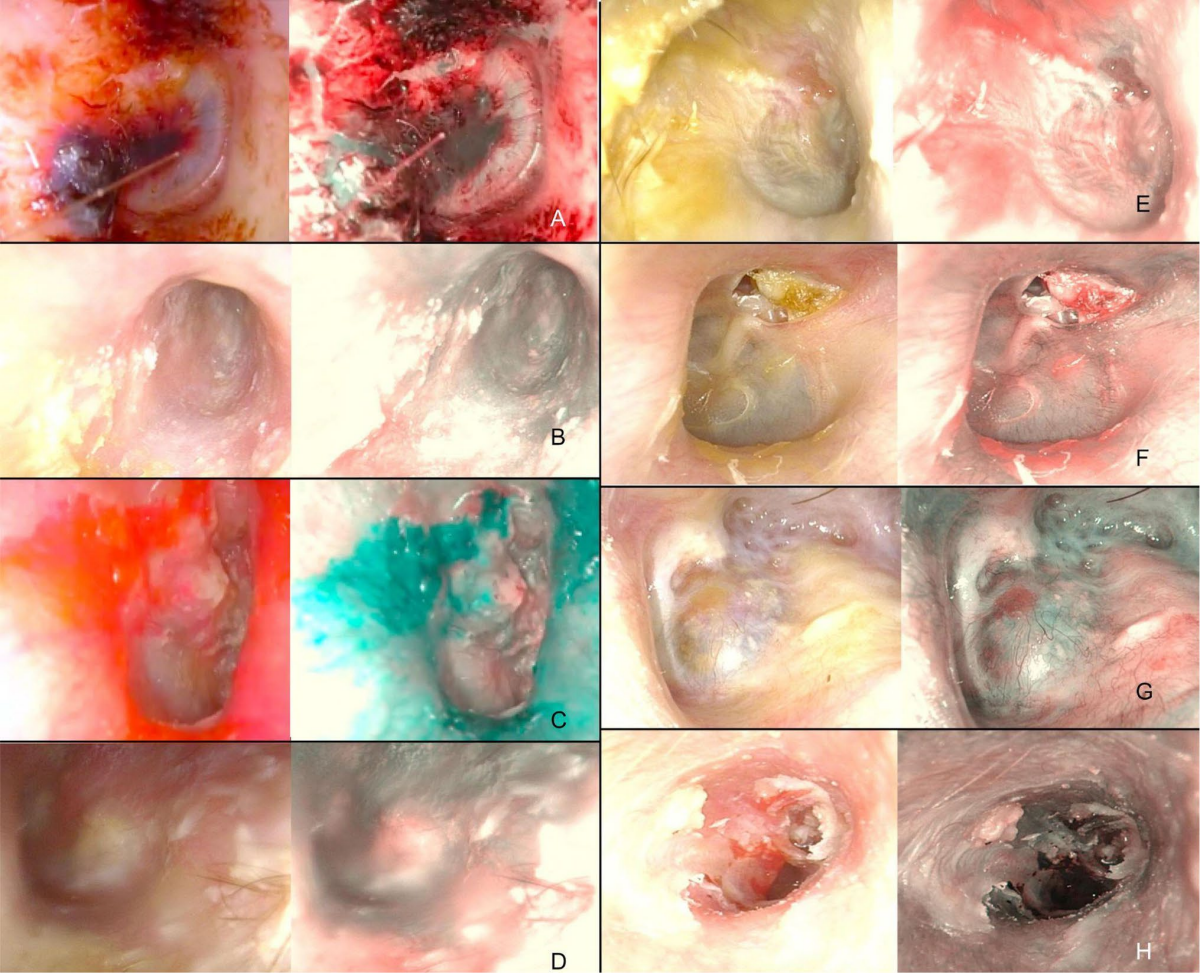
**A** Bullous myringitis; **B** bacterial otitis externa; **C** otomycosis; **D** acute otitis media; **E** granular myringitis; **F** cholesteatoma; **G** secondary tympanomastoid cholesterol granuloma; **H** attic retraction pocket with the presence of a cholesteatomatous matrix and a perforation of the pars tensa with granulations on the posterior margin

Bullous myringitis is characterized by the presence of hemorrhagic blebs on the tympanic membrane and on the skin of the external auditory canal. The blebs usually burst spontaneously between the outer and middle layers or toward both sides of the eardrum [19]. In Fig. 3a, we report the most representative one and, in all three cases, NBI simply highlighted the presence of coagulated blood material in the canal and hyperemia of the membrane in all cases.

Bacterial otitis externa is commonly caused by *P. aeruginosa* or *S. aureus*, and it is characterized by purulent exudate involving the ear canal [20]. A diffuse enhancement of vascularization was always appreciated by NBI, and this was probably favored by the general de-epithelialization of the canal. The eardrum always appeared completely purple–dark blue with the loss of otoscopic landmarks (Fig. 3B).

Otomycosis is a fungal infection (usually mediated by *A. niger* or *Candida *spp.) of the external ear that can manifest in both immunosuppressed and immunocompetent patients, as a consequence of a prolonged exposition to a heat and humid environment (e.g., swimming pool) and that is favored by other dermatological conditions, such as psoriasis or atopical dermatitis. This infection determines edema and erythema of the meatal epithelium of the auditory canal and tympanic membrane, with whitish cotton-like debris [21]. In these cases (Fig. 3C) NBI revealed a milder inflammatory reaction compared to bacterial cases and that appeared in a more limited and mottled diffusion in over 80% of cases.

For AOM cases, standard otoendoscopy revealed the classic hyperemic membrane, with signs of serous transudate coming from the tympanic cavity. It was also possible to appreciate the pulsation due to the inflammatory process in progress [22]. Under NBI the hyperemic eardrum area appeared deep purple, and it was possible to appreciate the presence of dark blue material at the lower level of the tympanic cavity (Fig. 3D). Finally, for the much rarer granular myringitis (whose granulations and de-epithelialization on the lateral surface of the tympanic membrane are not associated with middle-ear disease) [23]. In our experience, the use of NBI does not add anything to the standard otoscopy (Fig. 3E).

### Cholesteatoma

Cholesteatoma is characterized by a keratinizing stratified squamous epithelium, surrounded by an active matrix capable of releasing osteolytic enzymes [24]. In the study period, 9 cholesteatomas were studied with NBI (Fig. 3F) and they always retained the same pearly white appearance, a feature that allowed them to be clearly demarcated from the surrounding pinkish tissues, as it is also reported by other authors [6].

We also analyzed a case of left canal wall-down tympanoplasty for cholesteatomatous chronic otitis media with a secondary tympanomastoid cholesterol granuloma, histologically confirmed after revision surgery. A left retrotympanic bluish mass was noticed in otomicroscopy. At NBI the mass is more violet-like, without a pulsatile or bulging appearance, confirming the hematic origin of the disease, due to an anaerobic break-down of blood products for a negative middle ear pressure (Fig. 3G) [25, 26].

Finally, we report the case of a patient with an attic retraction pocket with the presence of a cholesteatomatous matrix and a perforation of the pars tensa with granulations on the posterior margin. As shown in Fig. 3h, all the features described above are well represented.

### Paragangliomas of the middle ear

Paragangliomas usually presented with a reddish-violet pulsatile mass behind an intact eardrum or in the posteroinferior part of the external auditory canal, with associated hearing loss and pulsatile tinnitus [27].

**Fig. 4:**
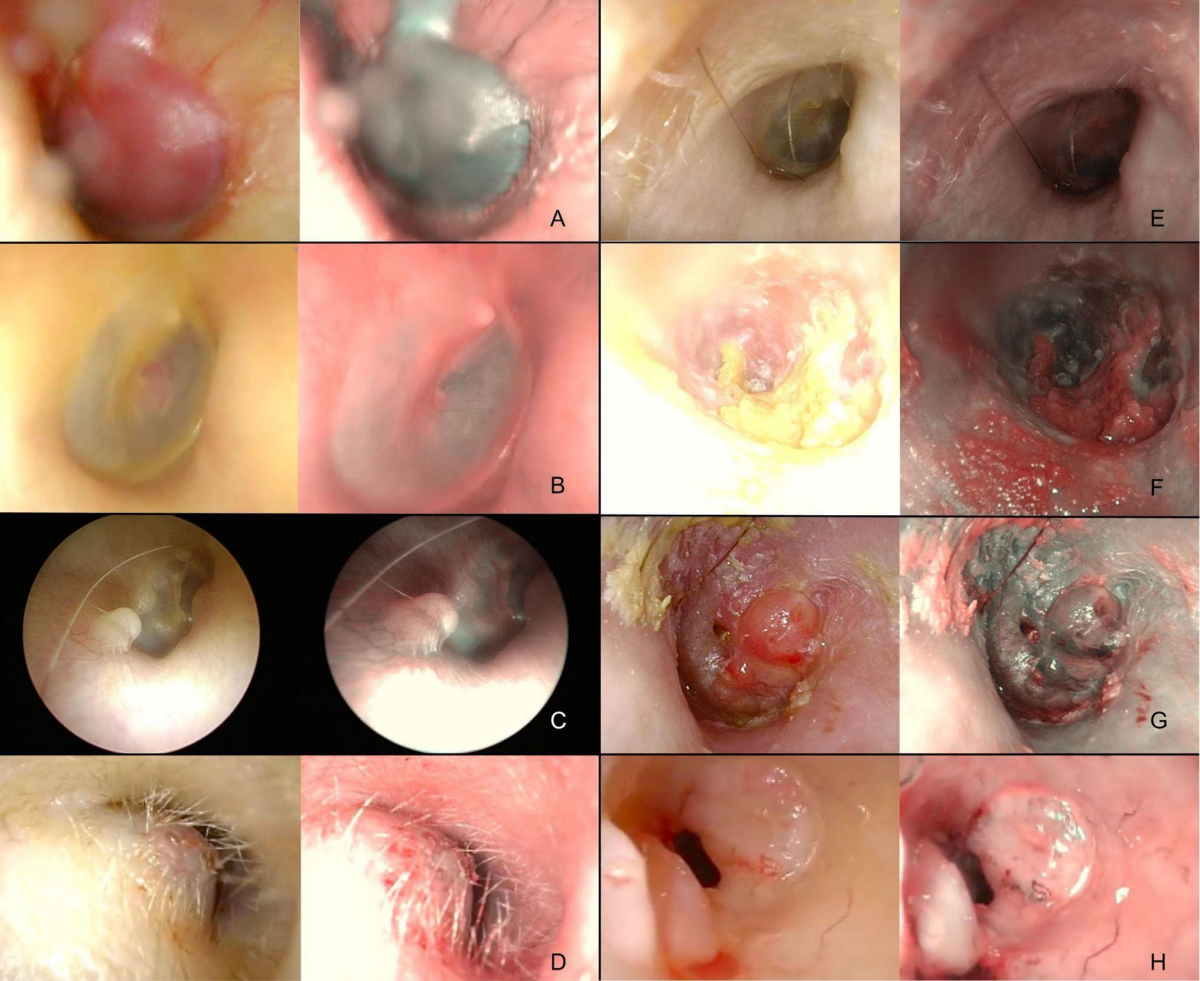
**A** Stage B paraganglioma; **B** stage A paraganglioma (localized at the promontory); **C** exostosis; **D** cavernous hemangioma; **E** syringocystadenoma of the external auditory canal; **F** locally advanced ceruminous adenocarcinomas of the external ear; **G** nasopharyngeal carcinoma with direct involvement of middle ear structures; **H** basal cell carcinoma

We analyzed 10 cases of paragangliomas with NBI, whose appearance was dependent on their clinical stage according to the Fisch–Mattox classification [28]. More advanced lesions (80% in our series, stage B or higher, and at least partially protruding toward the external ear canal) appeared as entirely uniform dark blue masses under NBI, and their local extension was more easily discernible compared to WL (Fig. 4A). On the contrary, for the two cases of Stage A mesotympanic paragangliomas, WL was paradoxically superior in revealing the mass because of the loss of membrane translucency with NBI (Fig. 4B).

### Benign and malignant tumors of the external ear canal

Benign tumors of the external ear canal are very rare, with osteomas being the most frequent histotype [29]. Osteomas are typically unilateral, while exostoses are bilateral sessile bony protuberances in the medial part of the external auditory canal that result from repetitive cold water exposure because of endosteal irritation and focal bone growth [30]. In our case series, we studied with NBI 17 cases of exostoses and osteomas of the external ear and in more than half of them (11/17, 70%) we noticed how the lesion overstretched the tiny capillaries that are perfectly visible. In Fig. 4C, a representative case is represented.

Regarding malignant tumors, we have described the appearance of one nasopharyngeal carcinoma with direct involvement of middle ear structures (Fig. 4G), one case of squamous cell carcinoma, and two cases of basal cell carcinoma of the canal (one of them is represented in Fig. 4H). The former showed a dark blue mass on NBI that lateralizes the tympanic membrane, with also a reactive polypoid component at the posterosuperior level (Fig. 4G). For all the primary cutaneous malignancies we noticed in all cases a clearer representation of the superficial vascular texture of the lesion along with its boundaries (Fig. 4H).

In our series, we also came across much rarer entities: a case of cavernous hemangioma (a benign vascular hamartomatous cystic lesion) [31], whose NBI appearance did not substantially differ from the white light appearance (Fig. 4D); an extremely rare case of a syringocystadenoma of the external auditory canal [32, 33], that presented as an isolated thickening with a single small verrucous papula on the anterior wall and that with NBI showed no vascularization (the subcutaneous glandular mass revealed on histopathology to have an overlying normal epidermis) (Fig. 4E); and finally, a case of locally advanced ceruminous adenocarcinomas of the external ear: the infiltrated eardrum appears diffusely thickened and has a diffuse purplish appearance (Fig. 4F) [34].

### Osteoradionecrosis of the outer ear

**Fig. 5:**
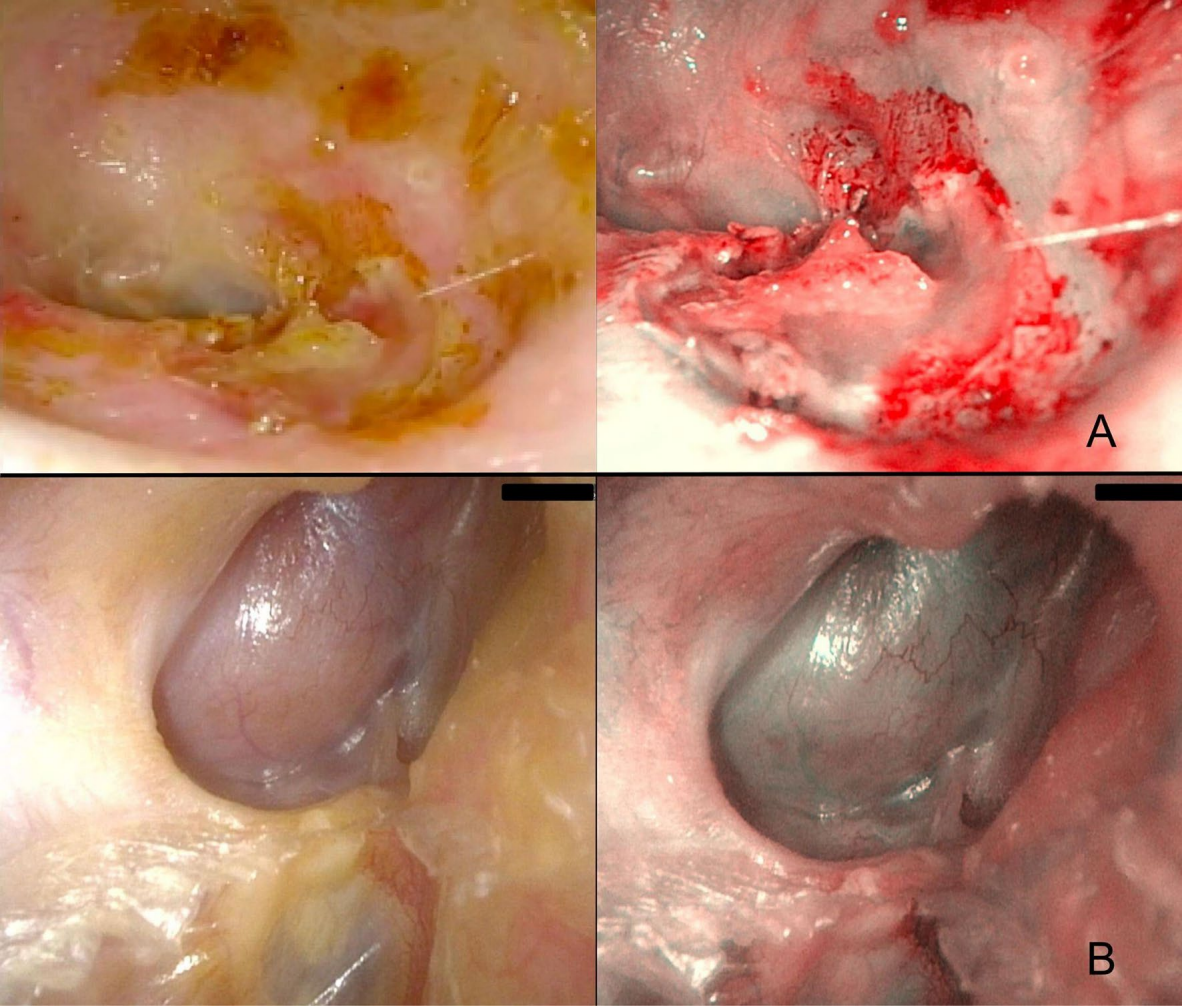
**A** Osteoradionecrosis; **B** meningoencephalic herniation

Osteoradionecrosis of the temporal bone can result in ulceration, epithelial thickening with stenosis, atrophy of cerumen glands, and cholesteatoma of the external auditory canal [35]. We have followed 3 cases of post-radiotherapy osteitis (all for nasopharyngeal carcinoma). The alterations revealed by both WL and NBI were always non-specific with an enhanced demarcation between atrophic and hyperemic areas (Fig. 5A).

### Tegmen tympani dehiscence

Meningoencephalic herniation usually occurs because of the dehiscence of the tegmen tympani or tegmen mastoideum [36]. In an emblematic case, reported in Fig. 5B, a rounded encapsulated mass in the most lateral part of the mastoid cavity was observed, together with a clear enhancement of the meningeal vessel under NBI.

## Discussion

NBI has gained much popularity in all fields of otorhinolaryngology, going from the characterization of nasal masses to the laryngopharyngeal reflux disease [37, 38] With our case series we have tried to illustrate the possible advantages and disadvantages of using NBI in various pathologies of the external and middle ear. Table 1 gives a schematic overview of the pros and cons of enhanced otoendoscopy for each condition.

It was immediately evident to us that this method does not provide additional information compared to traditional otomicroscopy in disorders characterized by poor vascularization (retraction, myringosclerosis, etc.) or subcutaneous disorders (osteoma, exostosis). Interestingly, even for an overtly vascular lesion, such as ear canal hemangioma, NBI was not helpful in the preoperative diagnosis, because the overlying intact skin prevented the direct visualization of its texture.

For inflammatory ear disorders, this technique may actually highlight the true extent of the pathology, but it does not provide any information on the involvement of the middle ear. It also did not discriminate the etiology of the otitic process, while the purulent secretions, debris, or the hyphal material in the canal very often impair a correct evaluation of the vascular patterns. Because in the standard otoscopic appearance of AOM the vascularization/”redness” of the membrane was found to be less important compared to other signs or symptoms (bulging, pain, etc.), it would be interesting to reevaluate this parameter by this technique [39].

Instead, narrow band imaging might play a prognostic and diagnostic role in the evaluation of acute tympanic perforations, paragangliomas, and epithelial neoplasms.

It is known that acute tympanic perforations, whatever their etiology, are more likely to spontaneously close if they are small and with bleeding margins[40]. We can speculate that cases of tympanic perforation where NBI show a visible capillary structure of the tympanum may be associated with more favorable outcomes, and we are planning to verify this hypothesis in the near future.

NBI may also play a role in the differential diagnosis of vascular masses, such as paragangliomas [12]. We analyzed four cases of tympanic/jugular glomus and in three of them, NBI confirmed its utility to define the vascular aspect of the lesions, giving a more complete view of the extent of the pathology at least in the tympanic cavity. In the last case, conversely, the small glomus of the promontory was better visualized in otomicroscopy. The reason may be related to the fact that the paraganglioma was not in direct contact with the tympanic membrane and that the presence of air between these two structures can somehow affect the resolution of the NBI images. In the cases of malignant epithelial tumors, NBI demonstrated a generic superficial vascular pattern, reflective of the neoangiogenesis typical of neoplastic formations. A specific pattern that may differentiate one histology from another was not identified but the number of analyzed cases is too small before drawing any conclusion on this aspect.

A limitation of the present work is represented by the small number of patients included because of the many discarded cases. As in other districts, a clean field of view is necessary to appreciate the vascular patterns and indeed cerumen removal and ear canal manipulation represent a great limitation. Another problem comes from the correlation between the NBI appearance and the non-histologically confirmed conditions: despite a diagnostic bias may well be present, this is a purely explorative research paper, so the sensitivity, specificity, and accuracy of the technique were not evaluated at all. Finally, we have to recall that there are also other optical methods that have been applied for the otological diagnosis: for instance, optical coherence tomography has been shown to outperform the diagnostic accuracy of simple otoscopy for AOM [41, 42]. Furthermore, in the near future deep learning methods might even substitute human evaluation for AOM or COM, and they can be implemented using a smartphone-connected otoscope [43, 44]. Despite this exciting progress, a formal comparison between these diagnostic methods, and sound reproducible evidence in the current otological practice is still lacking.

## Conclusion

In the present pictorial review, we have illustrated the role of contemporary otoendoscopic diagnosis with the aid of an enhanced imaging technique. NBI has potentially useful applications in the differential diagnosis of some external and middle ear masses, while for other disorders, its role seems much less important. Formal head-to-head diagnostic studies must be nonetheless conducted to give this technique clinical relevance.

**Table 1:** Pros and cons of the use of NBI during otoendoscopic evaluation of several external or middle ear disorders

Outer/middle ear pathology	*N*° of cases	PROS	CONS
Tympanic perforation	30	The pattern may represent a prognostic factor for any spontaneous closure or engraftment of the temporalis fascia after myringoplasty, emphasizing the tympanic vascular texture	None
Atrophic areas, pars tensa retractions, myringosclerosis	62	None	None
Infectious external/middle ear disorders	41	It may enhance the inflammatory aspect of otitis	It does not determine the true extent of the pathology, even at the level of the middle ear
Cholesteatoma	9	It may aid in the differentiation of the white matrix from the surrounding pink tissues	None
Tympanic/jugular paraganglioma	10	It may raise the suspicion the pathology in the tympanic cavity	There is a poor resolution in case of promontory lesion due to the air present between the glomus and the eardrum
Osteoradionecrosis of the temporal bone	3	None	None
Benign tumors			
Osteomas	17	None	None
Cavernous hemangioma	1	If overlying skin is not too thick, it gives excellent visualization of the angiomatous lesion	If subcutaneous, it provides almost no information
Benign ceruminous gland neoplasms	1	None	None
Malignant tumors			
Adenocarcinomas	1	It may emphasize the surrounding inflammatory skin	It does not provide information on the extension of the neoplasm
Nasopharyngeal carcinoma	1		
Squamous cell carcinoma	1	It may provide a good visualization of the superficial neoangiogenesis	
Basal cell carcinoma	2		
Tegmen tympani dehiscence	1	It may give a better visualization of meningeal vessels	None
